# The clinical effects of cerebral near-infrared spectroscopy monitoring (NIRS) versus no monitoring: a protocol for a systematic review with meta-analysis and trial sequential analysis

**DOI:** 10.1186/s13643-021-01660-2

**Published:** 2021-04-16

**Authors:** Mathias Lühr Hansen, Simon Hyttel-Sørensen, Janus Christian Jakobsen, Christian Gluud, Elisabeth M. W. Kooi, Jonathan Mintzer, Willem P. de Boode, Monica Fumagalli, Ana Alarcon, Thomas Alderliesten, Gorm Greisen

**Affiliations:** 1 Department of Neonatology, Rigshospitalet, Copenhagen University Hospital, Blegdamsvej 9, 2100 Copenhagen, Denmark; 2 Department of Intensive Care, Rigshospitalet, Copenhagen University Hospital, Blegdamsvej 9, 2100 Copenhagen, Denmark; 3 Copenhagen Trial Unit, Centre for Clinical Intervention Research, Rigshospitalet, Copenhagen University Hospital, Blegdamsvej 9, 2100 Copenhagen, The Capital Region of Denmark Denmark; 4 Department of Regional Health Research, The Faculty of Health Sciences, University of Southern Denmark, Odense, Denmark; 5 Division of Neonatology, Beatrix Children’s Hospital, University Medical Center Groningen, University of Groningen, Groningen, Netherlands; 6 Department of Pediatrics, Division of Newborn Medicine, Mountainside Medical Center, Montclair, NJ USA; 7 Division of Neonatology, Department of Pediatrics, Radboud University Medical Center, Radboud Institute for Health Sciences, Amalia Children’s Hospital, Nijmegen, Netherlands; 8 Fondazione IRCCS Ca’ Granda Ospedale Maggiore Policlinico Milan, Via Francesc Sforza 35, 20122 Milano, Italy; 9 Department of Clinical Sciences and Community Health, University of Milan, Via Festa del Perdono 7, 20122 Milano, Italy; 10 Department of Neonatology, Hospital Sant Joan de Deu, Passeig de Sant Joan de Deu 2, 08950 Esplugues de Llobregat, Barcelona, Spain; 11 Department of Neonatology, University Medical Center Utrecht Brain Center, Utrecht University, Utrecht, Netherlands

**Keywords:** Systematic review, Cerebral NIRS monitoring, Cerebral oxygenation monitoring, Hypoxic-ischaemic brain injury, Intensive care, Anaesthesia, Surgery, Neonatal intensive care, Meta-analysis, Trial sequential analysis

## Abstract

**Background:**

Multiple clinical conditions are associated with cerebral hypoxia/ischaemia and thereby an increased risk of hypoxic-ischaemic brain injury. Cerebral near-infrared spectroscopy monitoring (NIRS) is a tool to monitor brain oxygenation and perfusion, and the clinical uptake of NIRS has expanded over recent years. Specifically, NIRS is used in the neonatal, paediatric, and adult perioperative and intensive care settings. However, the available literature suggests that clinical benefits and harms of cerebral NIRS monitoring are uncertain. As rates of clinically significant hypoxic-ischaemic brain injuries are typically low, it is difficult for randomised clinical trials to capture a sufficiently large number of events to evaluate the clinical effect of cerebral NIRS monitoring, when focusing on specific clinical settings. The aim of this systematic review will be to evaluate the benefits and harms of clinical care with access to cerebral NIRS monitoring versus clinical care without cerebral NIRS monitoring in children and adults across all clinical settings.

**Methods:**

We will conduct a systematic review with meta-analysis and trial sequential analysis. We will only include randomised clinical trials. The primary outcomes are all-cause mortality, moderate or severe persistent cognitive or neurological deficit, and proportion of participants with one or more serious adverse events. We will search CENTRAL, EMBASE, MEDLINE, and the Science Citation Index Expanded from their inception and onwards. Two reviewers will independently screen all citations, full-text articles, and extract data. The risk of bias will be appraised using the Cochrane risk of bias tool version 2.0. If feasible, we will conduct both random-effects meta-analysis and fixed-effect meta-analysis of outcome data. Additional analysis will be conducted to explore the potential sources of heterogeneity (e.g. risk of bias, clinical setting).

**Discussion:**

As we include trials across multiple clinical settings, there is an increased probability of reaching a sufficient information size. However, heterogeneity between the included trials may impair our ability to interpret results to specific clinical settings. In this situation, we may have to depend on subgroup analyses with inherent increased risks of type I and II errors.

**Systematic review registration:**

PROSPERO CRD42020202986. This systematic review protocol has been submitted for registration in the International Prospective Register of Systematic Reviews (PROSPERO) (http://www.crd.york.ac.uk/prospero) on the 12th of October 2020 and published on the 12th of November 2020 (registration ID CRD42020202986).

**Supplementary Information:**

The online version contains supplementary material available at 10.1186/s13643-021-01660-2.

## Background

### Description of the condition

The brain depends on a constant supply of nutrients and oxygen for structural integrity and function. Different mechanisms such as low blood pressure, inadequate cardiac output, hypoxaemia, and hypocapnia (decreased carbon dioxide in the blood) may lead to inadequate blood supply to the brain (hypoxia/ischaemia) [1–4] and ultimately to brain injury [3–7]. Typical patients with hypoxic-ischaemic brain injury are those with stroke, those resuscitated after cardiac arrest, those rescued from severe casualties, those with perinatal asphyxia, or those with critical cardiopulmonary organ failure [8–12].

Loss of consciousness occurs in seconds after cardiac arrest and injurious processes from lack of oxygen occur within minutes [13]. In drowning or choking, this process may take longer due to the reservoir of oxygen in the pulmonary alveoli [14]. There are differences between the loss of respiration and oxygen supply and the loss of circulation and delivery of oxygen to tissues. However, from the cellular perspective, the common effect is a critical reduction in local tissue oxygen concentration, reduced mitochondrial oxidative phosphorylation, reduced intracellular ATP concentration, reduced Na-K pumping across the plasma membrane, loss of membrane potential, and from there on a range of destructive (and reparative) processes starts with resultant tissue damage [13].

Brain injury, defined as the acute reaction to a hypoxic-ischaemic insult can be diagnosed by neurological examination, electrophysiology methods, or functional tests [15, 16]. Brain injury may lead to brain damage that can be diagnosed by brain imaging [17–19]. Brain damage may lead to more or less permanent neurological or cognitive impairment, depending on location, extent, and compensatory mechanisms [12, 19]. While brain imaging can be used to detect brain damage and define clinically relevant outcomes, later neurological examinations, functional testing, or questionnaires can be used to define relevant patient outcomes. Full recovery may occur both in children and adults, while more subtle impairment may only become evident with long-term follow-up — which especially holds true in newborns.

### Clinical settings with elevated risk of hypoxic-ischaemic brain injury

#### Anaesthesia and surgery

Cerebral oxygenation may drop due to hypoxaemia, hyperventilation, and/or hypotension [20–23]. This often happens to a mild degree but may be severe due to complications of anaesthesia or the surgical procedure itself. Cardiac surgery under cardiac arrest or during cardiopulmonary bypass carries specific risks for cerebral oxygenation impairment as well. General recovery after anaesthesia and surgery can be protracted, may involve an element of cognitive dysfunction, and occasionally frank neurological deficit appears [24–27].

#### Intensive care

Critical illness with respiratory failure or circulatory shock is associated with brain injury or dysfunction [28, 29]. Mental and cognitive recovery after intensive care can be protracted, with occasionally unexpected neurological deficits. Current research is focusing on pathogenesis, prediction, and prevention of these outcomes [30–32].

#### Preterm birth

Newborns adapt to extra-uterine life during the first minutes, hours, and days of life [33]. Filling of the lungs with air, establishing respiration and pulmonary blood flow, and closing of the foramen ovale and the ductus arteriosus all constitute components of this transition [4]. In preterm neonates, this challenge is compounded by immature lungs, inadequate surfactant production, and myocardial immaturity with limited diastolic as well as systolic function [4]. In extremely preterm infants, this process often requires cardiorespiratory support, and in spite of this, neurodevelopmental impairment is common [34].

### Monitoring of cerebral oxygenation

Direct monitoring of cerebral oxygenation is not routine during general anaesthesia [35] or in the intensive care unit [36, 37]. However, clinical uptake is growing, especially during cardiac and aortic surgery [38–40] and in paediatric [36] and neonatal intensive care [37]. Several cerebral oxygenation monitoring methods are available: jugular venous blood sampling or catheter-based oximetry to measure cerebro-venous oxygen saturation, oxygen electrodes to measure tissue oxygen tension, or near-infrared spectroscopy (NIRS) to measure mixed arterio-venous (‘tissue’) oxygen saturation [41]. Cerebral NIRS has the distinct advantage of being non-invasive, theoretically with few adverse effects, and is applicable in almost all patients [42].

### The theoretical pros and cons of monitoring cerebral oxygenation

The question is, if monitoring of cerebral oxygenation has added value for the patient, i.e. if it contributes to a better clinical outcome when compared with routine monitoring [43]. As the pathophysiology of hypoxic/ischaemic brain injury is well established, safeguarding brain oxygenation should facilitate primary prevention of brain injury. The delivery of oxygen to tissue depends on the interplay of many factors, such as the reactions of cerebral vasculature to hypotension and hypoxia, as well as the oxygen carrying capacity of the blood, haemoglobin affinity for oxygen, and blood viscosity [44, 45]. The oxygen balance in tissue also depends on oxygen demand that may be increased in fever or during seizures, among other conditions [46].

On the other hand, measurements of cerebral oxygenation are not always accurate and may sometimes be erroneous [47, 48], and thresholds for intervention may be poorly defined [49]. Moreover, monitoring entails risks of overtreatment and undertreatment. For example, if FiO_2_ is unnecessarily increased due to hypoxic cerebral oxygenation values, an increased risk of bronchopulmonary dysplasia and retinopathy of prematurity in preterm infants may follow [50, 51]. In the SafeBoosC-II trial, comparing treatment guided by cerebral NIRS monitoring versus treatment and monitoring as usual in extremely preterm infants, an increased incidence of bronchopulmonary dysplasia and retinopathy of prematurity was seen in the experimental group [52]. Monitoring also has its costs in terms of equipment and clinicians’ time and attention.

### Randomised clinical trials of a complex intervention

The gold standard for testing the benefits and harms of diagnostic tests (NIRS) and following treatments (reactions to improve cerebral oxygenation) is randomised clinical trials [53, 54]. The diagnostic method must be connected to an intervention to produce a clinical benefit. For a monitoring technique such as cerebral NIRS monitoring, in addition to the complexities of defining thresholds for action, as well as defining the range of potential interventions, there is also the issue of time. It is thus difficult precisely to define the intervention that is tested in a trial assessing the effects of NIRS, and randomised clinical trials will depend on the clinical skills of the clinicians caring for patients in the experimental as well as in the control group. Potentially, the control group can be monitored, but with a blinded screen, which allows retrospective analysis of options for brain-dedicated interventions.

### Previous evidence on the use of cerebral NIRS monitoring in different clinical settings

According to our knowledge, three systematic reviews with meta-analysis have evaluated the use of cerebral NIRS monitoring to guide clinical care compared to clinical care without access to cerebral NIRS monitoring, in different clinical settings, with a goal of promoting neuroprotection [38, 39, 55].

One review evaluated the use of cerebral NIRS monitoring in patients undergoing cardiopulmonary bypass [38]. It included 1466 adults across ten trials and found no significant difference in mortality or stroke between experimental and control participants [38]. However, the number of events were low. The number of deaths in the experimental group were 6/298 participants versus 10/310 participants in the control group. For strokes, the numbers were 9/567 participants in the experimental group versus 9/571 participants in the control group [38]. The review did not use sequential methods (e.g. trial sequential analysis) to control risks of type I and type II errors [56]. Furthermore, several of the included trials were assessed at high risk of bias due to unclear information on blinding of clinical personnel, participants, and outcome assessors, insufficient reporting of data completeness for the primary outcome, and selective reporting bias with only one trial having published a protocol. The GRADE assessment showed low or very low certainty of evidence for all outcomes [38]. Thus, the authors concluded that the existing evidence does not indicate a significant clinical benefit of cerebral NIRS monitoring during cardiac surgery and that there is a need for additional, low risk of bias randomised clinical trials to determine the clinical effect of the intervention [38].

A Cochrane review from 2018 also evaluated the use of cerebral NIRS monitoring in the perioperative setting [39]. This review included randomised clinical trials evaluating cerebral NIRS monitoring during all types of surgery. The review included 1822 adult participants across 15 trials. No significant differences were found between experimental and control participants, for either mortality, postoperative stroke, postoperative delirium, or postoperative cognitive dysfunction. However, as with the systematic review on cardiopulmonary bypass [38], the number of events were low, and only one to six trials were included in the meta-analysis for each outcome (1/195 versus 2/195 deaths, 1/120 versus 4/120 postoperative strokes, 9/94 versus 14/96 episodes of postoperative delirium, and 13/24 versus 27/33 episodes of postoperative cognitive dysfunction after one week of surgery, in the experimental and control group) [39]. This review also did not use sequential methods (e.g. trial sequential analysis) to control risks of type I and type II errors [56]. The authors graded the certainty of evidence as low to moderate, mainly due to uncertainty regarding allocation concealment, potential conflict of interest from industry sponsorship, unclear blinding status, missing participant data, and imprecision. Based on the review results, the authors conclude that it is uncertain whether cerebral NIRS has a clinical benefit as a perioperative monitoring tool, due to low quality of evidence as well as low number of events [39].

An additional Cochrane review from 2017 evaluated the use of cerebral NIRS monitoring in very preterm infants [55]. The review included only one randomised clinical trial with a total of 166 participants and found no significant differences between the experimental and control participants for neither mortality (12/86 versus 20/80), mild-moderate brain injury by ultrasound (49/80 versus 33/77), or severe brain injury by ultrasound (10/80 versus 18/77) [55]. The trial, however, was not powered to detect a relevant difference on any of these clinical outcomes. Due to the small sample size, lack of group allocation blinding, and the surrogate outcomes being indirectly linked to the clinical outcomes, the authors graded the certainty of evidence as very low to low. The review authors concluded that, based on the available evidence, the effect of cerebral NIRS monitoring in preterm infants cannot be established and that there is a need for additional large-scale randomised clinical trials with clinically relevant primary outcomes [55].

The characteristics of these three reviews are summarized in Table 1.

**Table 1:** Overview of previous systematic reviews and meta-analyses evaluating the effect of clinical care guided by cerebral NIRS monitoring on neurological outcomes

First author	Title	Study design	Intervention/comparison	Primary (and neurological) outcome(s)	No. of trials	No. of participant	Published/registered protocol	Adverse events	Risk of bias	Conclusion
Serraino et al. [38]	Effects of cerebral near-infrared spectroscopy on the outcome of patients undergoing cardiac surgery	Systematic review and meta-analysis	Cerebral NIRS monitoring compared with no cerebral NIRS monitoring or an alternative goal-directed therapy	Mortality, acute brain injury (stroke or TCI), neurocognitive function, S100B levels	10	1466 (adults)	Yes	No	Yes	Existing evidence shows no effect of the intervention on clinical outcomes. More RCTs at low risk of bias are needed.
Yu et al. [39]	Cerebral near-infrared spectroscopy (NIRS) for perioperative monitoring of brain oxygenation	Cochrane review	Cerebral NIRS monitoring compared with blinded or no cerebral NIRS monitoring for perioperative monitoring of brain oxygenation in children and adults	Mortality, postop. stroke/adverse neurodev. outcomes, POD/POCD	15	1822 (adults)	Yes	Yes	Yes	The effect of the intervention is uncertain due to low quality of evidence. More RCTs are needed, especially in the paediatric population, since no such trials exist outside the neonatal care.
Hyttel-Sørensen et al. [55]	Cerebral near-infrared spectroscopy monitoring for prevention of brain injury in very preterm infants	Cochrane review	Cerebral NIRS monitoring compared with blinded or no cerebral monitoring for at least 24 h in very preterm infants	Mortality, neurodev. disability, IVH, cPVL	1	166 (preterm infants)	Yes	Yes	Yes	Based on one trial with a surrogate primary outcome, the systematic review did not reach sufficient power to prove or disprove the interventions effect on clinical outcomes. More RCTs are needed.

*cPVL* cystic periventricular leukomalacia, *IVH* intraventricular haemorrhage, *NIRS* near-infrared spectroscopy, *RCTs* randomised clinical trials, *POCD* postoperative cognitive decline, *POD* postoperative delirium, *TCI* transient cerebral ischaemia

### The motivation and aim of the present meta-analysis

The primary purpose of monitoring of cerebral oxygenation is the prevention of hypoxic/ischaemic brain injury. The rates of clinically significant hypoxic/ischaemic brain injury, however, are typically low, and large numbers of participants are needed to exclude effects of importance [56]. Therefore, the purpose of this systematic review with meta-analysis and trial sequential analysis is to evaluate the beneficial and harmful effects of clinical care with access to cerebral NIRS monitoring versus clinical care without access to cerebral NIRS monitoring, in children and adults across all clinical settings. Since the primary purpose of NIRS monitoring is prevention of brain injury, the majority of the chosen outcomes focuses on this.

## Methods

This protocol is in adherence with the Preferred Reporting Items for Systematic reviews and Meta-Analyses for Protocols (PRISMA-P) statement [57, 58] (see checklist in Additional file 1). This protocol has been registered within the International Prospective Register of Systematic Reviews (PROSPERO) database (registration ID CRD42020202986) [59, 60].

### Eligibility criteria

Studies will be selected according to the following criteria.

#### Types of studies

We will include randomised clinical trials, irrespectively of publication status, publication type, publication year, or written language. Cluster randomised trials and the first part, before cross-over, of randomised cross-over trials will also be included. Quasi-randomised studies and other studies that are not randomised clinical trials will be excluded.

#### Types of participants

We will include adults and children of all ages, including neonates, irrespectively of sex and comorbidities.

#### Types of interventions

The experimental intervention will be cerebral NIRS monitoring to guide clinical care, irrespectively of the length of the intervention period and clinical setting. The control intervention will be no access to cerebral NIRS monitoring. In some trials, participants in the control group will have received cerebral NIRS monitoring to collect data on cerebral oxygenation values during the trial, but where the oxygenation values were unavailable to the clinical staff. In such trials, the control intervention will also be defined as no access to cerebral NIRS monitoring, as this additional monitoring to collect data, was not a part of the control intervention.

Any co-interventions can be accepted but only if the co-intervention is planned to be delivered similarly in both the experimental and control group.

### Outcomes

#### Primary outcomes

1. All-cause mortality at maximal follow-up.
2. Moderate or severe, persistent cognitive or neurological deficit, significantly affecting daily life, at maximum follow-up (e.g. modified Rankin score of three or higher [61], Gross Motor Function Classification System level two or higher [62], or Bayley Scale of Infant Development score below minus two standard deviations at 2 years or later [63]). If several of such outcomes are reported, then we will choose the outcome with the highest proportion reported in each trial.
3. Proportion of participants with one or more serious adverse events. We will define a serious adverse event as any untoward medical occurrence that resulted in either death, was life-threatening, jeopardised the participant, was persistent, led to significant disability, led to hospitalisation, or led to prolonged hospitalisation [64]. As we expect the trialists’ reporting of serious adverse events to be heterogeneous and not strictly according to the International Committee of Harmonization-Good Clinical Practice (ICH-GCP) recommendations, we will include the event as a serious adverse, if the trialist either: (a) used the term ‘serious adverse event’ but not refer to ICH-GCP [64] or (b) reported the proportion of participants with an event we consider to full-fill the ICH-GCP definition (e.g. myocardial infarction or hospitalisation). If several of such outcomes are reported, then we will choose the outcome with the highest proportion reported in each trial.

#### Secondary outcomes

1. Mild, moderate or severe, temporary or persistent, cognitive or neurological deficit as defined by the trialists (e.g. postoperative delirium, postoperative cognitive decline, or Bayley Scale of Infant Development score below minus one standard deviations [63]). If several of such outcomes are reported, then we will choose the outcome with the highest proportion reported in each trial.
2. Quality of life defined as any validated continuous outcome scale used by the trialists at maximum follow-up.
3. Any evidence of brain damage on imaging as defined by the trialists at maximal follow-up. If several of such outcomes are reported, then we will choose the outcome with the highest proportion reported in each trial.
4. Proportion of participants with one or more adverse events defined as an untoward medical occurrence that did not necessarily have had to have a causal relationship with the intervention and which is also non-serious [64].

#### Exploratory outcomes

1. Any evidence of a negative impact on the brain as defined by the trialists (including mild, moderate or severe, temporary or persistent cognitive or neurological deficits, evidence of brain damage on imaging, or evidence of brain damage on electrophysiological monitoring). If several of such outcomes are reported, then we will choose the outcome with the highest proportion reported in each trial.
2. Individual serious adverse events [64]
3. Individual adverse events [64]

### Information sources and search strategy

Trials will be identified through systematic searches within the following databases, from inception and onwards: Cochrane Central Register of Controlled Trials (CENTRAL), EMBASE, MEDLINE, and Science Citation Index Expanded. No language or time restriction will be applied. The reference lists of all relevant trials will be checked as well.

A detailed search strategy for MEDLINE Ovid as well as the search results can be found in ‘Additional file 2’. The search strategy will be adapted to other databases.

### Searching other resources

As an additional search tool, we will search for unpublished and ongoing trials at clinicaltrials.gov and the following medical device companies websites: Medtronic, Minneapolis, MN, USA; NIRO, Hamamatsu, Hamamatsu City, Japan; CAS Medical, Branford, CT, USA; Nonin Medical, Plymouth, MN, USA; Masimo, Irvine, CA, USA; Enginmed, Suzhou, China; and Oxyprem, Zürich, Switzerland. Furthermore, we will also identify ongoing relevant trials through trial registers in Europe and USA including clinicaltrials.gov.

For ClinicalTrials.gov, we will conduct the following search:

Condition or disease: (Near-infrared spectroscop*) OR (Near-infrared spectromet*) OR (Near infrared spectroscop*) OR (NIR spectromet*) OR (NIR spectroscop*) OR (NIRS) OR (Oxygenati*) OR (Oxygen saturation) OR (Oximetr*) OR (Cerebral perfusion) OR (Cerebral saturation)

Other terms: (Randomized) or (Randomised)

Study type: Interventional Studies (Clinical trials)

Study results: all studies

### Data collection and analysis

#### Selection of studies

All trials that are identified during the literature search will be uploaded to EndNote (Clarivate, Philadelphia, PA, USA). Two authors (MLH and SHS) will screen the titles and abstracts of the identified studies. If a study is deemed potentially relevant by either one of the two authors, the full text will be retrieved and assessed for eligibility by the same two authors. If ineligible, the reason for exclusion will be documented. If there is a disagreement between the two authors regarding eligibility or ineligibility, a third author (JCJ) will make the final decision of inclusion or exclusion. Eligible trials will be included in the systematic review and a Preferred Reporting Items for Systematic Reviews and Meta-Analysis (PRISMA) flow diagram will be included as well. Also, a table displaying the characteristics of excluded studies will be presented in the final systematic review [65]. Analysis of cluster randomised trials and the first part, before cross-over, of randomised cross-over trials, will be handled as depicted in The Cochrane Handbook for Systematic Reviews of Interventions [66].

#### Data extraction and management

Once the relevant trials have been included, data extraction will be conducted by the two authors MLH and SHS independently. Any disagreements will be discussed in the author group and a final decision will be made. The following data will be extracted from each study:

1. General information: title, author(s), year of publication, language of publication, funding sources, and potential conflicts of interest
2. Methodology: study design, clinical setting, inclusion and exclusion criteria, type of interventions, cerebral NIRS monitoring unavailable for clinical staff in the control group, outcome measures, and time of outcome assessment
3. Sample size: number of participants meeting the criteria for inclusion

We will use specific data extraction forms designed for this purpose. If some of the relevant data is not available in the study report or publication, e.g. if the study does not report all of the pre-specified outcomes, the trialists will be contacted and asked if they can provide such data. The correspondence with the trialists will be included in the systematic review as an appendix.

#### Outcome classification

The two authors MLH and SHS will present all the relevant, extracted outcome measures from each included trial to the authors GG and JCJ, without revealing the number of outcome events in the experimental and control group. GG and JCJ will then classify the outcomes according to the primary, secondary, and exploratory outcome definitions in this protocol. If there is disagreement between GG and JCJ on any outcome classification, a fifth author (CG) will make the final decision.

### Assessment of risk of bias in included studies

Randomised clinical trials with certain methodological flaws carry an increased risk of bias [67–72]. Such methodological flaws increase the likelihood that the trialists will come to the wrong conclusion by over- or underestimating effect sizes [73]. Therefore, it is important to assess the risk of bias in trials included in a systematic review [65]. Based on the Cochrane risk of bias tool — version 2 (RoB 2) [74] described in The Cochrane Handbook for Systematic Reviews of Interventions [75], we will assess the risk of bias for the following domains: (1) bias arising from the randomisation process, (2) bias due to deviation from intended interventions, (3) bias due to missing outcome data, (4) bias in measurements of outcomes, and (5) bias in selection of the reported results. Risk of bias assessment of the included studies will be conducted by the two authors MLH and SHS, who independently will transfer data into the Stata file. Any disagreement between their assessments will be discussed and, if necessary, a final decision will be made by a third author (JCJ).

#### Bias arising from the randomisation process

*A trial will be considered at low risk of bias* if the allocation sequence was adequately concealed (e.g. performed by an on-site locked computer, a central independent unit or sealed, identical envelopes), *and* there are no baseline imbalances between the experimental and control group (if any appeared, they must be compatible with chance), *and* the allocation sequence generation was adequate (e.g. generated by a computer random numbers generator, a random numbers table, tossing a coin, shuffling cards or drawing lots - the latter three methods will only be considered low risk of bias if the sequence generation was conducted by an independent person with no involvement in the trial), *or* if a description of the method for allocation sequence generation is missing.

*A trial will be considered of some concerns* if the allocation sequence was adequately concealed *and* there is a problem with the method of allocation sequence generation, *or* baseline imbalances suggest a problem with the randomisation process, *or* if the method for allocation concealment was not described for the trial, *and* baseline imbalances across intervention groups appear to be by chance, *or* if no information is available to answer any of the signaling questions.

*A trial will be considered at high risk of bias* if investigators were aware of the allocation sequence [68], *or* if the method for allocation concealment was not described, *and* if baseline imbalance suggest a problem with the randomisation process.

Furthermore, trials where the generation was not at random, or quasi-randomised, will be considered high risk of bias and excluded from the review [71, 76]

#### Bias due to deviation from intended interventions

*A trial will be considered at low risk of bias* if participants and clinical staff — and parents in paediatric trials — were unaware of the group allocation during the trial, *or* if they were aware of the group allocation during the trial, but any deviation from the intended intervention reflected normal clinical practice, *or* if they were aware of group allocation, but any deviation from the intended intervention was unlikely to influence the outcomes, *and* no trial participants were analysed on the basis of the received intervention, instead of on the basis of their randomised allocation group.

*A trial will be considered of some concerns* if participants and clinical staff — and parents in paediatric trials — were aware of group allocation, *and* no information is available regarding deviations from normal clinical practice, which potentially could impact the outcomes *and* the deviations from clinical practice were imbalanced between the intervention groups, *or* some trial participants were analysed on the basis of the received intervention instead of on the basis of randomised allocation group, but it was deemed as insufficient to significantly alter the intervention effect estimate.

*A trial will be considered at high risk of bias* if participants and clinical staff — and parents in paediatric trials — were aware of group allocation [73, 77], *and* there were deviations from intended interventions which were unbalanced between the intervention groups, *and* likely to affect the outcomes, *or* some participants were analysed on the basis of the received intervention instead of on the basis of randomised group allocation, *and* it was deemed as sufficient to significantly alter the intervention effect estimate.

#### Bias due to missing outcome data

*A trial will be considered at low risk of bias* if there is no missing outcome data, *or* if the proportion of missing outcome data is similar between the intervention groups, *and* the reasons for missing outcome data are similar, *or* if there is evidence that the missing outcomes do not make an important difference to the estimate of the intervention effect (e.g. sensitivity analyses such as ‘best-worst, worst-best’ case scenario analysis).

*A trial will be considered of some concerns* if the amount of missing outcome data is unclear, *or* there is unclear information regarding the proportion of missing data between intervention groups, *and* reason for missing outcome data between intervention groups is unclear, *and* there is no evidence that the missing outcome data do not make an important difference to the estimate of the interventions effect (e.g. lack of sensitivity analyses such as ‘best-worst, worst-best’ case scenario analysis).

*A trial will be considered at high risk of bias* if the amount of missing data is high (more than 5%), *and* missing outcome data between the intervention groups differ, *or* the reason for missing outcome data between intervention groups differ, *and* there is no evidence that the missing outcome data do not make an important difference to the estimate of the interventions effect (e.g. lack of sensitivity analyses such as ‘best-worst, worst-best’ case scenario analysis) [71].

#### Bias in measurement of outcomes

*A trial will be considered at low risk of bias* if the outcome assessors were blinded to group allocation, *or* if the outcome assessors were not blinded to group allocation, but it was deemed that knowledge of group allocation was unlikely to influence outcome assessment.

*A trial will be considered of some concerns* if there is no available information to evaluate whether outcome assessors were blinded to group allocation *and* if such knowledge could influence outcome assessment.

*A trial will be considered at high risk of bias* if outcome assessors were not blinded to group allocation *and* it is deemed likely that knowledge of group allocation was likely to influence outcome assessment [73, 78].

#### Bias in selection of the reported result

*A trial will be considered at low risk of bias* if the outcome data reported are unlikely to have been selected based on the results of multiple outcome measurements (e.g. different scales to measure the outcome, multiple assessors of the outcome, different time points for assessment of the outcome) within the outcome domain, *and* if the outcome data reported are unlikely to have been selected based on the results from multiple outcome analysis.

*A trial will be considered of some concerns* if it is uncertain whether the outcome data reported have been selected based on the results of multiple outcome measurements (e.g. different scales to measure the outcome, multiple assessors of the outcome, different time points for assessment of the outcome) within the outcome domain *or* from multiple outcome analysis.

*A trial will be considered at high risk of bias* if the reported outcome data are likely to have been selected based on the results of multiple outcome measurements (e.g. different scales to measure the outcome, multiple assessors of the outcome, different time points for assessment of the outcome) within the outcome domain *or* from multiple outcome analysis.

### Overall risk of bias

The included trials will be considered as overall in low risk of bias or high risk of bias. A trial will be considered overall low risk of bias if the trial is judged as low risk of bias in *all* the above domains. If the trial is considered at high risk of bias, or to be of some concern, in any of the above domains, the trial will be considered as overall in high risk of bias. Within each trial, each outcome result will be assessed for bias, based on the three domains ‘bias due to missing outcome data’, ‘bias in measurements of outcomes’, and ‘bias in selection of the reported result’. Thus, we will be able to assess not only risk of bias in each trial, but also for each outcome. Additionally, the Grading of Recommendations, Assessment, Development and Evaluation (GRADE) assessment will be used to assess the quality of the body of evidence for all outcomes and summarized in a summary of findings table [79] (see the section ‘Summary of findings table’ for a description of the five considerations included in the GRADE assessment).

The primary conclusion will be based on the analysis of our primary outcome results in all trials assessed as having an overall low risk of bias [56].

### Assessment of bias in conducting the systematic review

The systematic review will be conducted according to this protocol. Any deviation in the conduct will be reported in the section ‘Differences in the methodology between protocol and review’ in the systematic review.

### Measures of treatment effect

#### Dichotomous outcomes

For dichotomous outcomes we will calculate risk ratios (RRs) with 95% confidence intervals (CIs) and trial sequential analysis-adjusted CIs [56, 80].

#### Continuous outcomes

For continuous outcomes, i.e. ‘Quality of Life’, we will calculate the standardized mean difference with a 95% CI and a trial sequential analysis-adjusted CI [56, 80].

#### Handling missing data

We will use the intention-to-treat data from the included trials for both dichotomous and continuous outcomes. For trials with missing or unclear outcome data, the trial authors will be contacted by MLH with JCJ as ‘cc’. The trial authors will be requested to provide missing outcome data or to elaborate on unclear outcome data. All correspondence will be attached to the systematic review in an appendix. If it is not possible to obtain missing outcome data, we will not impute the missing data for the primary analysis. Instead, this will be done in the sensitivity analyses.

### Data synthesis

A descriptive table featuring the included trials’ key characteristics and methodology will be reported, including the following information: title, author(s), year of publication, language of publication, funding sources, potential conflicts of interest, study design, clinical setting, inclusion criteria, exclusion criteria, types of intervention, outcome assessment, time of outcome assessment, and protocol status. All data analyses will be conducted in STATA 16.1 (StataCorp LLC, College Station, Texas, USA). The meta-analysis will be conducted as recommended in the Cochrane Handbook for Systematic Reviews of Interventions [81]. For outcomes where data is only available from one trial, the results will be narratively described. If one or more of the included trials reports on multiple intervention arms, we will only include the relevant arms. Furthermore, the population in the control group will be halved for such studies, if two of the comparisons are included in the meta-analysis. An eight-step procedure by Jakobsen et al. will be used to assess if thresholds for statistical and clinical significance are crossed [56].

#### Step one — meta-analysis

Both fixed-effect and random-effects meta-analyses will be used to estimate the effect of the intervention [82, 83]. The most conservative results (highest *P* value) will primarily be used and the less conservative result will be used as sensitivity analysis [56].

#### Step two — assessment of heterogeneity

Statistical heterogeneity will be evaluated by using *I*^2^ statistics, with a threshold for significant heterogeneity at *p* < 0.1 [84], and by visual inspection of forest plots. Clinical heterogeneity will be assessed by evaluating the characteristics of the included trials based on the PICO model (Participants, Interventions, Comparisons, Outcomes). Any signs of heterogeneity will be explored in the subgroup analyses.

#### Step three — accounting for multiplicity

Since we report on three primary outcomes, a *p* value below 0.025 will be considered statistically significant for each of the primary outcomes [56].

#### Step four — trial sequential analysis

To control the risks of type I and II errors [85], all primary outcomes will undergo trial sequential analysis and the required meta-analysis information size as well trial sequential boundaries for benefit, harm, and futility will be established [86]. If the required number of randomised participants to achieve sufficient power is not reached, the confidence interval for the point estimates will be adjusted accordingly by the trial sequential analysis program [80, 86]. A relative risk reduction of 20% will be used as the anticipated intervention effect for each primary outcome, an alpha of 2.5% will be used as the acceptable risk of type 1 errors and a beta of 10% will be used as the acceptable risk of type 2 errors.

For cumulative *Z*-scores that reach below 50% of the diversity-adjusted trial sequential analysis required information size (or sample size), we will downgrade imprecision by two levels for the GRADE assessments (see section on ‘Summary of findings table’). For cumulative *Z*-scores that reach between 50 and 100% of the diversity-adjusted trial sequential analysis required information size (or sample size), we will downgrade imprecision by one level for the GRADE assessments. For cumulative *Z*-scores that cross the monitoring boundaries for benefit, futility, or harm, we will not downgrade imprecision for the GRADE assessments.

#### Step five — Bayes factor

The Bayes factor [87] will be calculated for all primary outcomes and 0.1 will be used as threshold for significance [56]. An anticipated risk reduction of 20% will be used when calculating the Bayes factor [56].

#### Step six — subgroup and sensitivity analysis

The following subgroup analyses will be conducted, if possible:

1. Comparison of the intervention effect between trials at overall low to high risk of bias
2. Comparison of the intervention effect between trials assessing different clinical settings: neonatal intensive care, paediatric intensive care, children during surgery, adult intensive care, and adults during surgery
3. Comparison of trials without support from the medical device industry compared to trials at risk of such support [69]
4. Comparison of trials where participants in the control group underwent cerebral NIRS monitoring, but where the oxygenation values were unavailable to the clinical staff, compared to trials where participants in the control group did not undergo cerebral NIRS monitoring at all

To quantify the potential impact of missing outcome data, the following two sensitivity analysis will be conducted on the three primary outcomes:

- ‘Best-worst case’ scenario: we will assume that all participants lost to follow-up in the experimental group either died, suffered from ‘moderate or severe persistent cognitive or neurological deficit’, or had ‘one or more serious adverse events’, while all participants lost to follow-up in the control group experienced these events.
- ‘Worst-best case’ scenario: we will assume that all participants lost to follow-up in the experimental group suffered died, suffered from ‘moderate or severe persistent cognitive or neurological deficit’, or had ‘one or more serious adverse events’, while all participants lost to follow-up in the experimental group did not experience any of these events.

#### Step seven — assessment of risk of publication bias

If at least ten trials are included in the meta-analysis, we will create funnel plots and visually inspect them to assess any potential publication bias. As an additional measure, we will evaluate the funnel plot asymmetry by conducting the Harbord test [88] for dichotomous outcomes and the Egger test for continuous outcome [89].

#### Step eight — assessment of clinical significance

If the data analyses show statistically significant effects of the intervention, we will also assess whether the results are clinically significant. The assessment of clinical significance will be based on the definitions of minimal important differences (see ‘Step four — trial sequential analysis’) and the summary of findings table (see section on ‘Summary of findings table’) as well as a thorough evaluation of beneficial and harmful outcomes. Furthermore, we will calculate the number-needed-to-treat for all dichotomised outcomes [56].

### Summary of findings table

To present the findings for the pre-specified primary, secondary and exploratory outcomes, we will create and include a ‘Summary of findings’ table in the systematic review as recommended in the Cochrane Handbook for Systematic Reviews of Interventions [90]. The Grading of Recommendations, Assessment, Development and Evaluations (GRADE) approach will be used to assess and rate the quality of the body of evidence, i.e. the certainty in the range of an effect estimate for all pre-specified outcomes [79]. The GRADE approach evaluates the body of evidence based on the five following considerations: (1) risk of bias assessment [74, 91], (2) heterogeneity or inconsistency of results [92], (3) imprecision of the effect estimates due to wide CIs [93], (4) indirectness of evidence [94], and (5) publication and for-profit bias [95]. Imprecision will be assessed using trial sequential analysis [56]. To assess for-profit bias, we will search for information regarding industry funding for each trial and trial author. If a trial, or an author, is sponsored by the industry, we will judge the trial as in high risk of for-profit bias. To conduct the rating, we will use the online tool GRADEpro software (www.gradepro.org). The reasons for any up- or downgrading of the certainty of evidence will be described and justified in details in the systematic review [90].

As we expect heterogeneity due to the various clinical settings and wide age span of participants in the included studies within this systematic review, we will also create summary of findings tables for the subgroup analysis within each of the different clinical settings (e.g. neonatal intensive care trials, paediatric intensive care trials, adult intensive care trials, and perioperative care trials).

## Discussion

### Strengths

The present review has several methodological strengths. First, the methodology is predefined and registered on PROSPERO before any literature search has been conducted, since this decreases the risk of selective outcome reporting bias of the systematic review [96, 97]. Any changes to the protocol originating during the peer-review or systematic review process will be described in the systematic review in the section ‘Differences in the methodology between protocol and review.’ Second, the protocol includes a thorough description of the methodology which relies on the principles and methods outlined in The Cochrane Handbook for Systematic Reviews of Interventional Research, including a risk of bias assessment of all included studies as a tool to consider systematic errors [98]. Third, we plan to use the eight-step procedure by Jakobsen et al. to assess statistical and clinical significance for each of the pre-specified outcomes, which increases the validity of the systematic review [56]. The eight-step procedure includes Trial Sequential Analyses, which controls for random errors by checking if a sufficient information size has been reached [80] and whether the boundaries for futility, benefit, or harm have been crossed [86]. These analyses will assist us in determining the need for downgrading due to imprecision. To increase transparency and heighten the quality of the systematic review, we will also provide a section, outlining any deviation to this protocol, in the final publication.

### Limitations

Our review also has important methodological limitations. During the last decade, clinical research of NIRS monitoring has increased rapidly, primarily during surgery but also in the intensive care setting [38, 39, 55]. Parallel to this, the clinical uptake of NIRS monitoring has grown as well [36, 37]. As all randomised clinical trials evaluating the use of cerebral NIRS monitoring to guide clinical care, irrespectively of age or clinical setting, are eligible for this systematic review, we expect to include trials across various clinical fields. Since the population and study setting will then differ between included trials, there is a potential for both statistical and clinical heterogeneity. We will therefore conduct subgroup analyses on the different populations and clinical settings, e.g. subgroup analyses on studies within neonatal intensive care, paediatric intensive care, adult intensive care, and perioperative monitoring during surgery. Another limitation is our broad outcome definitions. Due to the expected heterogeneity in the included trials and to obtain sufficient information size for the meta-analysis, it is necessary to be pragmatic and, thus, classify and pool the outcomes reported in the included trials into broader outcomes (e.g. moderate or severe, persistent cognitive or neurological deficit, significantly affecting daily life). As the two authors, MLH and SHS, who will conduct the data extraction, will be aware of the number of events in the experimental and control group for each outcome in the included trials, there is a risk of outcome assessment bias, if they were to classify the trial outcome measures according to the definitions in this protocol. To minimise this risk of bias, the classification of trial outcome measures will be conducted by two authors (GG and JCJ) that have not taken part in the data extraction (see section on ‘Outcome classification’). If there is disagreement between GG and JCJ, a fifth author (CG) will make the final decision. As neither GG, JCJ, or CG have read the manuscripts or seen any data from the included trials, they will be blinded to group allocation, i.e. without knowledge of the number of outcome events in the experimental and control group.

Another limitation of this review is the potential lack of ability to directly affect clinical decision making. The primary analysis will rely on all included trials, irrespective of clinical setting or population. This increases the probability of reaching a sufficient information size [80]. Due to the expectedly large clinical heterogeneity between included trials, however, it will be difficult to interpret and extrapolate the results of the primary analysis directly to a specific clinical setting. This issue will be less of a problem in the subgroup analyses, but it is unlikely that we will reach a sufficient information size for these analyses, thus making it difficult to interpret the results due to high risks of type I and II errors [80].

If the analysis reveals that the intervention has a beneficial effect on any of the pre-specified outcomes, it will rather serve as an encouragement to continue the conduct of randomised clinical trials within each clinical setting, in order to reach a sufficient information size.

To disseminate this study, we intend to submit the results in one single manuscript, to a major international medical journal. Furthermore, we intend to submit the abstract for presentation at an international conference within neonatal intensive care, as the authors are based in this field.

## Supplementary Information

**Additional file 1:.** PRISMA-P 2015 Checklist.

**Additional file 2:.** Search strategy in MEDLINE Ovid and Results.

## Data Availability

All data generated or analysed will be included in the systematic review.

## Abbreviations

CI: Confidence interval
GRADE: Grading of Recommendations, Assessment, Development and Evaluations
ICH-GCP: International Council for Harmonisation of Technical Requirements for Pharmaceuticals for Human Use Good Clinical Practice
NIRS: Near-infrared spectroscopy
PRISMA: Preferred Reporting Items for Systematic Reviews and Meta-Analysis
PRISMA-P: Preferred Reporting Items for Systematic Reviews and Meta-Analysis Protocols
PROSPERO: Prospective Register of Systematic Reviews
RoB 2: Risk of bias tool 2
RR: Risk ratio
